# The Safety and Efficacy of a Therapeutic Vaccine for Chronic Hepatitis B: A Follow-Up Study of Phase III Clinical Trial

**DOI:** 10.3390/vaccines10010045

**Published:** 2021-12-30

**Authors:** Sheikh Mohammad Fazle Akbar, Mamun Al Mahtab, Julio Cesar Aguilar, Osamu Yoshida, Sakirul Khan, Eduardo Penton, Guillen Nieto Gerardo, Yoichi Hiasa

**Affiliations:** 1 Department of Gastroenterology and Metabology, Ehime University Graduate School of Medicine, Toon 791-0295, Japan; yoshidao@m.ehime-u.ac.jp (O.Y.); hiasa@m.ehime-u.ac.jp (Y.H.); 2 Department of Hepatology, Bangabandhu Sheikh Mujib Medical University (BSMMU), Dhaka 1000, Bangladesh; shwapnil@agni.com; 3 Center for Genetic Engineering and Biotechnology, Havana 10600, Cuba; julio.aguilar@cigb.edu.cu (J.C.A.); eduardopenton45@gmail.com (E.P.); gerardo.garcia@cigb.edu.cu (G.N.G.); 4 Department of Microbiology, Faculty of Medicine, Oita University, Oita 879-5593, Japan; sakirul@oita-u.ac.jp

**Keywords:** chronic hepatitis B, therapeutic vaccine, HBsAg/HBcAg vaccine, NASVAC, nasal vaccine, follow-up

## Abstract

The objective of the present study was to assess the safety and efficacy of a therapeutic vaccine containing both HBsAg and HBcAg (NASVAC) in patients with chronic hepatitis B (CHB) three years after the end of treatment (EOT) as a follow-up of a phase III clinical trial. NASVAC was administered ten times by the nasal route and five times by subcutaneous injection. A total of 59 patients with CHB were enrolled. Adverse events were not seen in any of the patients. Out of the 59 CHB patients, 54 patients exhibited a reduction in HBV DNA, compared with their basal levels. Although all the patients had alanine transaminase (ALT) above the upper limit of normal (>42 IU/L) before the commencement of therapy, the levels of ALT were within the ULN level in 42 patients. No patient developed cirrhosis of the liver. The present study, showing the safety and efficacy of NASVAC 3 years after the EOT, is the first to report follow-up data of an immune therapeutic agent against CHB. NASVAC represents a unique drug against CHB that is safe, of finite duration, can be administered by the nasal route, is capable of reducing HBV DNA and normalizing ALT, and contains hepatic fibrosis.

## 1. Introduction

Infection with hepatitis B virus (HBV) is one of the most complex global public health problems. HBV spreads when blood, semen, or other body fluids from an HBV-infected person enter the body of someone who is not infected. After entering a host, the HBV may be controlled by the host immune system, and the hosts exhibit no features of ongoing HBV replication and liver damages. A second scenario indicates that HBV-infected subjects show components of acute self-limiting resolving hepatitis. They usually develop life-long immunity against future HBV infection after resolving acute hepatitis B. About 1.7 billion people of the world follow either of these two pathways, as about 2 billion of the world’s population has been infected by HBV at some point in their life. Although there are some particular concerns about their management in the context of blood transfusion and liver transplantation, almost 1.7 billion HBV-infected people of these two scenarios do not represent an emergent public health nightmare [1]. In addition, a potent vaccine against HBV is available and a global drive for hepatitis B vaccination has contained new HBV infections considerably, in developed countries as well as in developing and resource-constrained countries [2]. The main problem with HBV infection is chronic HBV infection in a global context. Chronic HBV infection can be classified into the following five phases: (1) HBeAg-positive chronic infection, (2) HBeAg-positive chronic hepatitis, (3) HBeAg-negative chronic infection, (4) HBeAg-negative chronic hepatitis, and (5) HBsAg-negative phase [3]. The presence of HBsAg, the surface protein of HBV, in the sera indicates that there is ongoing replication of HBV. Another important antigen of HBV is hepatitis B core antigen (HBcAg), and immunity to this antigen bears both diagnostic and therapeutic implications. An estimated 296 million chronic HBV-infected people are the permanent and living reservoir of HBV, and they may transmit HBV infection to healthy persons. Moreover, about 12–25% of the 296 million chronic HBV-infected patients would also present evidence of liver damage in addition to harboring HBV DNA and HBsAg [4]. These patients are regarded as having chronic hepatitis (CHB), and considerable proportions of CHB patients are prone to develop severe complications such as cirrhosis of the liver (LC) and hepatocellular carcinoma (HCC). Both LC and HCC cause considerable compromise regarding the quality of life and substantial mortalities. In 2019, about 820,000 people died due to CHB-related liver diseases [4].

In 2015, the World Health Organization (WHO) planned to eliminate viral hepatitis as a significant public health threat by 2030. The program has been regarded as the “Elimination of Hepatitis by 2030”. One of the primary targets of this global approach is to bring 80% of CHB patients under treatment by 2030. Most liberal estimates indicate that as of 2020, only 5 million CHB patients are under some therapeutic regimen globally, whereas about 35–75 million CHB patients should be treated by 2030. Most of the CHB patients receiving some form of treatment are from advanced, prosperous, and developed countries. A minor proportion of CHB patients of developing and resource-constrained countries receive any therapy for CHB. However, more than 80–90% of CHB patients reside in these countries of Asia and Africa [5].

As of today, the following two groups of drugs are used for the treatment of CHB patients: interferons (IFNs; standard and its pegylated forms) and nucleos(t)ide analogs (NUCs) [6]. Due to the inherent limitations of IFNs regarding safety, cost, route of administration, and limited efficacy, IFNs could not be popularized, even in developed countries [7]. With the advent of NUCs, considerable optimism about the therapy of CHB was originated as NUCs can be given by the oral route and the drugs are comparatively cheaper. In addition, NUCs induced a reduction in and negativity of HBV DNA in considerable numbers of CHB patients, and some studies reported antifibrotic and anticancer effects of NUCs in some patients [8]. It seems that NUCs are effective as long as these are taken by the patients; however, these create major problems if NUCs are discontinued [9].

Thus, there is a pressing need to develop new therapeutics for CHB, which will be safe, effective, and used for a finite duration. HBV is a non-cytopathic virus and is usually unable to induce the direct destruction of hepatocytes. Antigen-specific immunity seems to be protective in nature, and HBcAg-specific immunity seems to have a dominant role in this regard [10]. These facts led to the development of different immune therapeutic strategies against CHB from the 1990s. However, proper immune therapeutics for CHB are yet to surface. One of the significant limitations of the development of immune therapy for CHB is the design of therapeutic maneuvers. Most immune therapeutic studies have been conducted as pilot studies or observational studies. Although some immune therapies have reported encouraging outcomes, new immune therapeutics have not provided follow-up data [11,12,13]. Thus, immune therapy for CHB has been confined to laboratory interest, and proper modes of commercialization and registration have not been adopted. There is no immune therapy for CHB patients that have received consensus from the patients and physicians.

After analyzing the scopes and limitations of different modes of evolving immune therapy for CHB, we developed an immune therapeutic approach using two antigens of HBV; HBsAg and HBcAg (named NASVAC, Center for Genetic Engineering and Biotechnology, Havana, Cuba). A systematic model of NASVAC development led us to assess the efficacy of NASVAC in HBV transgenic mice (HBV TM) that expressed HBV DNA and HBsAg in the sera [14]. Both HBsAg and HBcAg induced HBV-specific humoral and cellular immunity in HBV TM, and these were followed by the negativity of HBsAg and the production of anti-HBs. Additionally, NASVAC induced cytotoxic T lymphocytes in splenocytes of NASVAC-immunized HBV TM and caused HBsAg negativity in NASVAC-immunized HBV TM [15]. Additionally, the safety and immunogenicity of NASVAC were ascertained by immunizing normal volunteers with NASVAC via the nasal route [16]. Subsequently, the safety, efficacy, and mechanism of action of NASVAC were evaluated by conducting a phase I/II clinical trial in CHB patients [17]. In this study, the NASVAC-induced activation of HBV-specific immunocytes and antigen-presenting dendritic cells was documented [17]. Thus, relevant preclinical and clinical studies exposed the immune-modulatory capacity of NASVAC. Finally, a phase III clinical trial ascertained the safety, antiviral potentiality, and liver-protecting capacity of NASVAC in CHB patients during the end of treatment (EOT) and 24 weeks after the EOT [18]. To assess the safety and potentiality of NASVAC as a new therapeutic, we have provided follow-up data of NASVAC-treated patients two years after the EOT [19].

The objective of the present study was to assess the safety, antiviral, and liver-protecting capacity of NASVAC three years after EOT as a follow-up of evaluation of a phase III clinical trial [18]. The accumulated data will provide scientific insights into the scopes and limitations of NASVAC as a new therapeutic against CHB.

## 2. Materials and Methods

### 2.1. Design of Clinical Trials (Phase I/II and III) with NASVAC in CHB Patients

**Figure 1:**
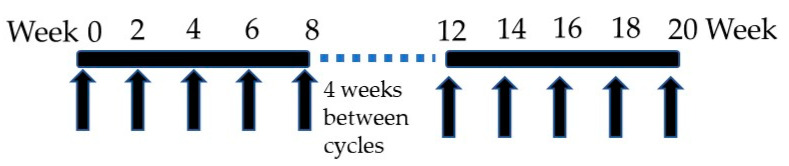
Schedule of Administration of NASVAC. NASVAC was given in two cycles. In the first cycle, the patients received NASVAC 5 times, once in two weeks, by intranasal administration. There was gap of 4 weeks between first and second cycles. In the second cycle, the patients received NASVAC both by intranasal and subcutaneous routes, 5 times.

NASVAC is a 1:1 formulation of 100 μg of HBsAg (Pichia pastoris-derived recombinant HBsAg subtype adw2) and 100 μg of HBcAg (purified Escherichia coli-expressed recombinant full-length HBcAg). It was derived from HBV genotype A. A phase I/II clinical trial was initially accomplished in 18 treatment-naïve CHB patients in Bangladesh in which NASVAC was administered 15 times (10 times by IN and 5 times by SC) (Figure 1). NASVAC was safe for all CHB patients, and also, its antiviral capacity (HBV DNA lowering or HBV DNA negativity) and liver-protecting potentials (normalization of ALT) were documented [17]. In addition, the exploration of the mechanism of action showed that NASVAC induced antigen-specific immunity in NASVAC recipients and activated antigen-presenting dendritic cells [17]. A phase III trial was conducted in 78 patients with CHB following exactly the same design as the Phase I/II clinical trial that administered NASVAC 15 times (Figure 1). Out of these 78 patients, HBV genotyping was conducted for 69 patients. The numbers of patients with HBV genotype A, C, and D were 20, 27, and 22, respectively.

The phase III clinical trial was conducted at Bangabandhu Sheikh Mujib Medical University, Dhaka, Bangladesh. A certain part of this phase III clinical trial was conducted at Farabi Hospital, Dhaka, Bangladesh. The ethics committee of Bangabandhu Sheikh Mujib Medical University, Dhaka, Bangladesh, approved the study, and the study was conducted in compliance with the Declaration of Helsinki. The principles of good medical practice were properly followed. The project identification code is NO. BSMMU/2010/2363, and the approval was given on 1 March 2010. The study was registered in ClincalTrials.Gov (NCT10374308). The patients were selected from a pool of CHB patients expressing at least 1000 copies/mL of HBV DNA and an elevated ALT above the upper limit of normal (ULN) [18]. The results indicate that 24 weeks after the EOT, NASVAC induced a significant reduction in HBV DNA and a normalization of ALT below ULN in considerable numbers of patients. Two years after EOT, a reduction in HBV DNA, and a normalization of ALT were found in considerable numbers of the patients studied [19].

### 2.2. The Present Study (Safety, Antiviral, and Liver Protection by NASVAC, 3 Years after EOT)

The main target of the present study was to follow up with patients of phase III clinical trials, as most of the immune therapies against CHB have not reported data about long-term follow-up. We communicated with all patients by phone and discussed their well-being. Although we contacted all 78 NASVAC recipients of the phase III trial, a total of 59 patients were finally enrolled in this study. These 59 patients attended hospitals as per permission of the institutional review board, and they also provided blood samples and undertook abdominal ultrasonography. Additionally, assessments of their ear, nose, and throat were performed three years after the EOT as NASVAC was administered via IN. They also attended a session with the principal investigator to assess their progression of liver fibrosis. The 19 patients who were not able to attend the principal investigator’s session at Dhaka, the capital of Bangladesh, for the 3-year follow-up did not report any adverse effects to the principal investigator of the study (Mamun Al Mahtab). The clinical profiles of the 59 patients are given in Table 1. The median age of the patient was 28 years (range; 18–50 years). Out of the total patients, 7 were female, and 52 were male. The levels of HBV DNA before the start of NASVAC therapy were more than 1.0 × 10^3^ copies/mL (range; 3.7 × 10^3^–1.0 × 10^13^). All patients had a serum ALT above the ULN level (>42 U/L) during the enrollment of the phase III clinical trial.

The patients who entered this follow-up were thoroughly checked for any adverse events during this period. Additionally, total blood counts were accomplished at the time of follow-up. In addition, serum hemoglobulin, creatinine, and bilirubin were checked in these patients. All patients were also checked by a specialist of ear, nose, and throat as NASVAC was given IN. Abdominal ultrasonography was conducted in these patients, and an upper gastrointestinal endoscopy was accomplished in suspected patients with CHB.

### 2.3. Quantification of Serum HBV DNA Levels

Serum HBV DNA was quantified in a polymerase chain reaction method using a commercial kit (Amplicon HBV Monitor Assay, RT-PCR, Roche Molecular Systems, Pleasanton, CA, USA). The lower limit of detection was 250 copies of HBV DNA/mL.

### 2.4. Assessment of ALT

One of the main purposes of this study was to provide uniform data 3 years after following up on the basis of initial data. To accomplish this, serum ALT of the present study was measured using a commercial kit approved by the hospital that provided the permission of the institutional review board.

### 2.5. Abdominal Ultrasonography and Upper Abdominal Endoscopy

Additionally, the same person performed the abdominal ultrasonography; however, he was not informed about the identity of the patients. Assessment of esophageal varices, if any, was also conducted by the specialized endoscopist.

## 3. Results

### 3.1. Long-Term Safety Concerns of NASVAC Recipients

NASVAC is a new type of therapeutic vaccine. It contained comparatively high levels of antigens (HBsAg, 100 µg; HBcAg, 100 µg). NASVAC is the first therapeutic vaccine licensed for the treatment of CHB and has been described extensively in previous communications. It is manufactured under good manufacturing practice (GMP) conditions. This product is a 1:1 formulation of 100 μg of HBsAg (Pichia pastoris-derived recombinant HBsAg subtype adw2) and 100 μg of HBcAg (purified Escherichia coli-expressed recombinant full-length HBcAg). These antigens were generated from HBV genotype A.

NASVAC is administered through intranasal (IN) and subcutaneous (SC) routes. The next is about the schedule of administration of NASVAC. It was administered ten times IN and five times SC. All the CHB patients were expressing HBV DNA and HBsAg in the blood with elevated ALT levels during enrollment in this clinical trial [18]. A thorough examination was accomplished to assess the safety of NASVAC 3 years after EOT. There were no subjective complaints regarding the usage of NASVAC. A total of 78 patients were included in the phase III clinical trial. The present study that was designed to evaluate safety and efficacy 3 years after EOT included 59 patients with CHB, as they satisfied the follow-up criteria. However, the safety issues were evaluated in all 78 patients via telephone communication. There was no evidence of inflammation of the nasal mucosa in any patient, as NASVAC was given by IN route. ENT specialists confirmed this result. The total counts of white blood cells were within the normal range, as shown in Table 2. The levels of bilirubin, albumin, and creatinine were also within the normal range in all patients. The ultrasonographic assessment also showed that the levels of hepatic fibrosis were between fibrosis levels one and two in all the patients. The parameters of the safety of NASVAC recipients are shown in Table 2.

### 3.2. Levels of HBV DNA in the Sera in NASVAC-Receiving CHB Patients 

The levels of HBV DNA in the sera varied considerably among the CHB patients. However, all of them had at least 1.0 × 10^3^ copies of HBV DNA in their sera, as per the inclusion criteria of this study. In some patients, the levels of HBV DNA in the sera were as high as 1.0 × 10^13^ copies/mL. Studies about immune therapy in CHB have reported notable effects of different immune therapeutic agents in CHB patients. However, investigators have mostly reported the antiviral effect at the EOT or within a short period after the EOT. This study was designed in such a way that the antiviral potentiality of NASVAC can be provided systematically. As mentioned, a total of 78 patients were enrolled in a phase III clinical trial. In this follow-up study, a total of 59 patients could be properly checked, and they were enrolled in a 3-year follow-up. Although all patients expressed more than 1000 copies of HBV DNA/mL at the start of therapy with NASVAC, HBV DNA was undetectable in 29 patients at the EOT. On the other hand, HBV DNA was below the level of detection in 20 patients 3 years after the EOT. However, we found that the levels of HBV DNA reduced in 54 CHB patients out of a total of 59 patients 3 years after the EOT, compared with the levels of HBV DNA at the basal levels (Table 3). Moreover, 14 patients remained negative for HBV DNA 24 weeks after the EOT, 2 years after the EOT, and 3 years after the EOT.

### 3.3. ALT Levels as Assessed Three Years after EOT

As mentioned in our first report about the treatment of CHB patients by NASVAC, all patients had elevated ALT levels when they were enrolled in the phase III clinical trial. The levels of ALT were measured again on the day of the first administration of NASVAC, as ALT usually shows marked fluctuations in CHB patients. A flare of hepatitis was seen at 12 weeks after commencement of therapy in most of the patients, as reported in the first report of the phase III clinical trial [18]. The levels of ALT were normalized or reduced in all patients by EOT. Afterward, a flare of ALT was not detected in these patients. At the end of 3 years after the EOT, the ALT levels were below ULN in 42 patients with CHB (Table 3). A total of 15 patients with CHB had ALT levels within two times the ULN level. Only two patients had ALT levels above two times the ULN level.

### 3.4. Antifibrotic Effect of NASVAC

All patients in this cohort were checked for progression of fibrosis in the liver three years after the EOT. The principal investigator took a detailed history of their illness, and they were subjected to abdominal ultrasonography and upper gastrointestinal endoscopy if needed or if any suspicion remained. There was no evidence of progression of liver fibrosis or the development of LC in any patient. Additionally, there were no HCC patients in this series.

## 4. Discussion

Two groups of antiviral drugs (IFNs and NUCs) have been used for more than two decades in patients with CHB. The inherent limitations of IFNs are related to their cost, safety concerns, route of administration, and limited efficacy. On the other hand, although NUCs offer an oral drug regimen, they should be used for an infinite duration or entire life, and the cessation of NUCs in CHB patients may result in a flare of hepatitis and life-threatening outcomes [6,7,8,9].

The fundamental aspect of CHB is related to the fact that the etiological agent of the disease, HBV, is a non-cytopathic virus. It is now evident that the impaired immunity that results from host/HBV interaction determines the nature of CHB-related pathogenesis. This is supported by the fact that about 90% of HBV-infected patients resolve their infection without any drug and pass an uneventful life. Although these factors point toward the development of immune therapy for CHB, there is no acceptable immune therapeutic regimen for CHB patients. However, several immune therapeutic drugs have been used for CHB patients during the last three decades, but none of them could stand the test of time [11,12,13]. This failure of the development of an immune therapy drug is not because of the low efficacy or increased adverse effects of immune therapeutic agents. Additionally, the efficacy of immune therapeutic agents for CHB did not play a vital role. This is mainly attributable to the improper study design and lack of professionalism regarding the establishment of immune therapy for CHB. Any innovative drug may have a promising or pessimistic outcome. However, there should be clinical trials with evidence-based medications. Proper follow-up data should be provided for a long time to decide whether the safety and efficacy is a short-lasting phenomenon, or this represents a sustained effect.

The present study is different from other immune therapeutic trials. It was originally planned to assess the safety and efficacy in long-term perspectives. The data documented here as outcomes of the follow-up data of 3 years after the EOT indicate that NASVAC is a safe immune therapeutic drug for CHB patients. It has passed all the major safety concerns by repeated consultation with the patients and also from the assessment of parameters of functions of vital organs. There was a significant concern if NASVAC induced any inflammatory changes in the nasal mucosa, as the drug was given by the IN route. An ENT specialist checked all the patients, and no such evidence was detected. Additionally, the levels of WBC, bilirubin, creatinine, and hemoglobulin were within the normal range at different points of the follow-up. We were also highly concerned about the effect of HBcAg in CHB patients. However, no flare of hepatitis was detected in any patients three years after the EOT. Taken together, NASVAC can be regarded as a safe drug.

Regarding the efficacy, NASVAC reduced the levels of HBV DNA in most patients, compared with their basal levels; however, the reduction in HBV DNA levels at the EOT and 3 years after the EOT provides an insight into the scope and limitation of immune therapy for CHB patients. However, out of the 59 patients in this cohort, HBV DNA was reduced in 54 patients, compared with their basal level. In addition, although all the patients had higher levels of ALT at the start of therapy, the levels of ALT reduced below ULN in 52 patients with CHB. Most importantly, none of the NASVAC-receiving CHB patients developed complications such as LC or HCC. These outcomes clearly reveal that a 3-year follow-up of a new and novel immune therapeutic drug may not be enough, and long-term follow-up would be required to have insights into the scope and limitation of any evolving therapy. The administration of NASVAC by the IN route is also an important point, as, on the one hand, this is patient friendly, and on the other hand, an IN administration induces mucosal immunity [20]. This also represents a patient-friendly attitude, as one of the notable limitations of the present study is the comparatively small sample size. The study began with 78 patients with CHB; however, 59 patients with CHB participated in the 3-year follow-up study. The next limitation is the lack of data on quantitative levels of HBsAg. However, quantitative HBsAg could not be measured in Bangladesh, where the clinical trial occurred. Thus, the assessment of HBsAg was not included in the original study design. However, a recent study in Japan estimated HBsAg in CHB patients treated with NASVAC. The study has shown that NASVAC is capable of reducing HBsAg in considerable numbers of CHB patients [21].

## 5. Conclusions

We documented the safety and efficacy of NASVAC as a new and novel immune therapeutic for CHB patients by systemic experimental approaches using good manufacturing practice (GMP). This is an evidence-based approach, as it contains HBcAg, and investigators have shown that HBcAg-specific immunity is essential for a reduction in HBV DNA and the normalization of ALT [10]. The GMP level of NASVAC was used from preclinical studies in HBV TM [14,15]. The phase I/II clinical trial data and the possible mechanism of action were provided before accomplishing the phase III clinical trial [17]. The phase III clinical trial unveiled its safety and efficacy at the EOT, and 24 weeks after the EOT [18]. The efficacy was also maintained 2 years after the EOT [16]. Finally, the present study showed that the antiviral and liver-protecting capacities of NASVAC were retained in considerable numbers of patients 3 years after the EOT, as compared with their basal levels. Thus, the necessity of a prolonged follow-up of 5 years or more would be required for optimizing an immune therapy for CHB. Thus, a finite therapeutic regimen of the immune therapeutic drug was shown for treating CHB patients with profound safety and moderate efficacy. Several options remain to accelerate the efficacy of NASVAC by alteration of the dose, the duration of therapy, and a combination with antiviral or other immune therapeutics. NASVAC is also friendly for patients of developing and resource-constrained countries, as this can be given by the IN route. The role of NASVAC should be considered in treating millions of CHB patients to attain the target of the “Elimination of Hepatitis B by 2030”, especially in developing and resource-constrained countries as a finite regimen of therapy for CHB patients.

## Figures and Tables

**Table 1:** Characteristics of the patients enrolled in this study on the day prior to administration of 1st dose of NASVAC.

Variables	Values
Total numbers	59
Male:Female	52:7
Age (Years)	28 (18–50)
HBV DNA (copies/mL)	4.9 × 10^4^ (Range; 1.7 × 10^3^–1.0 × 10^13^)
Alanine aminotransferase (IU/L) *	30 (10–262)
Level of fibrosis	F0–F2

* The values of ALT just prior to administration of 1st dose of NASVAC are shown.

**Table 2:** Safety profiles of NASVAC in patients with chronic hepatitis B at different points.

Variables	Basal Level	EOT	3 Years after EOT
White Blood (Counts/mm^3^)	9.0 (5.3–10)	9.0 (5.4–11)	9.0 (5.0–13.0)
Bilirubin (mg/dL)	0.63 (0.2–1.26)	0.7 (0.4–1.16)	0.68 (0.53–0.92)
Albumin (gm/dL)	3.7 (3.4–4.2)	4.1 (3.6–4.6)	4.78 (4.02–5.71)
Creatinine mg/dL)	0.99 (0.46–1.8)	1.0 (0.62–1.42)	1.01 (0.68–1.55)
Hemoglobulin (gm/dL)	13.2 (12.6–14.2)	12.4 (11.3–15.2)	13.8 (12–14.6)
Platelets count	230,000 (190,000–270,000)	210,000 (195,000–245,000)	220,000 (170,000–280,000)

Basal level: the day of 1st injection; EOT: end of treatment; 3 years after EOT: 3 years after the end of treatment during follow-up.

**Table 3:** Viral and biochemical data of the patients at three points.

Variables	Basal Level (N = 78)	EOT (N = 78)	3 Years after EOT (N = 59)
HBV DNA (log/copies/mL)	5.4 ± 2.1	2.8 ± 1.1	3.7 ± 1.7
HBV DNA (<250 copies.ml) (Number of Patients)	0	29 (37.2%)	20 (33.9%)
ALT (IU/L)	58.1 ± 29.5	40 ± 20.2	44.4 ± 20.6
ALT (<42 IU/L) (Numbers of patients)	0	55 (78.5%)	42 (88.1%)

Basal level: the day of 1st injection; EOT: end of treatment; 3 years after EOT: 3 years after the end of treatment during follow-up. For ALT, basal level indicates the level of ALT during final enrollment (within 3 months of administrating first dose of NASVAC). Data are shown as mean and standard deviation. Data in parentheses indicate percentage of that group.

## Data Availability

All data can be available from Mamun Al Mahtab, Head, Department of Hepatology, Bangabandhu Sheikh Mujib Medical University (BSMMU), Dhaka, Bangladesh (shwapnil@agni.com). He is the principal investigator of the study.

## References

[1] MacLachlan J.H., Cowie B.C. (2015). Hepatitis B virus epidemiology. Cold Spring Harb. Perspect. Med..

[2] Akbar S.M.F., Al Mahtab M., Begum F., Hossain S.A.S., Sarker S., Shrestha A., Khan M.S.I., Yoshida O., Hiasa Y. (2021). Implication of birth-dose vaccination against hepatitis B virus in South-east Asia. Vaccines.

[3] European Association for the Study of the Liver (2017). EASL 2017 Clinical Practice Guidelines on the management of hepatitis B virus infection. J. Hepatol..

[4] WHO (2017). Global Hepatitis Report 2017.

[5] Wahhed Y., Siddiq M., Jamil Z., Najmi M.H. (2018). Hepatitis elimination by 2030: Progress and challenges. World J. Gastroenterol..

[6] Terrault N.A., Bzowej N.H., Chang K.M., Hwang J.P., Jonas M.M., Murad M., American Association for the Study of Liver Diseases (2016). AASLD guidelines for treatment of chronic hepatitis B. Hepatology.

[7] Ozaras R., Khodor H., Yetim N., Unal U.K., Demirhan Y.E., Gultekin G., Isal B. (2015). Monotherapy for hepatitis B infection: A review of treatment options. Expert Rev. Anti-Infect. Ther..

[8] Wu J., Yin F., Zhou X. (2018). Efficacy of nucleoside analogues for hepatitis B virus-related liver failure: A network meta-analysis. Acta Pharm..

[9] Liem K.S., Fung S., Wong D.K., Yim C., Noureldin S., Chen J., Feld J.J., Hansen B.E., Janseen H.L. (2019). Limited sustained response after stopping nucleos(t)ide analogues in patients with chronic hepatitis B: Results from a randomised controlled trial (Toronto STOP study). Gut.

[10] Maini M.K., Boni C., Lee C.K., Larrubia J.R., Reignat S., Ogg G.S., King A.S., Herberg J., Gilson R., Alisa A. (2000). The role of virus-specific CD8(+) cells in liver damage and viral control during persistent hepatitis B virus infection. J. Exp. Med..

[11] Pol S., Driss F., Michel M.L., Nalpas B., Berthelot P., Brechot C. (1994). Specific vaccine therapy in chronic hepatitis B infection. Lancet.

[12] Boni C., Barili V., Acerbi G., Rossi M., Vecchi A., Laccabue D., Penna A., Missale G., Ferrari C., Fisicaro P. (2019). HBV immunetherapy: From molecular mechanisms to clinical applications. Int. J. Mol. Sci..

[13] Xu D.Z., Zhao K., Guo L.M., Li A.-L., Xie Q., Ren H., Zhang J.-M., Xu M., Wang H.-F., Huang W.-X. (2008). A randomized controlled phase IIb trial of antigen-antibody immunogenic complex therapeutic vaccine in chronic hepatitis B patients. PLoS ONE.

[14] Akbar S.M., Chen S., Al-Mahtab M., Abe M., Hiasa Y., Onji M. (2012). Strong and multi-antigen specific immunity by hepatitis B core antigen (HBcAg)-based vaccines in a murine model of chronic hepatitis B: HBcAg is a candidate for a therapeutic vaccine against hepatitis B virus. Antiviral Res..

[15] Akbar S.M., Yoshida O., Chen S., Cesar A.G., Abe M., Matsuura B., Hiasa Y., Onji M. (2010). Immune modulator and antiviral potential of dendritic cells pused with both hepatitis B surface antigen and core antigen for treating chronic HBV Infection. Antivir. Ther..

[16] Betancourt A.A., Delgado C.A., Estévez Z.C., Martinez J.C., Rios G.V., Aureoles-Rosello S.R.M., Guzman M.A., Baile N.F., Reyes P.A.D., Ruano L.O. (2007). Phase I clinical trial in healthy adults of a nasal vaccine candidate containing recombinant hepatitis B surface and core antigens. Int. J. Infect. Dis..

[17] Al-Mahtab M., Akbar S.M., Aguilar J.C., Uddin M.H., Khan M.S., Rahman S. (2013). Therapeutic potential of a combined hepatitis B virus surface and core antigen vaccine in patients with chronic hepatitis B. Hepatol. Int..

[18] Al Mahtab M., Akbar S.M.F., Aguilar J.C., Guillen G., Penton E., Tuero A., Yoshida O., Hiasa Y., Onji M. (2018). Treatment of chronic hepatitis B naïve patients with a therapeutic vaccine containing HBs and HBc antigens (a randomized, open and treatment-controlled phase III clinical trial). PLoS ONE.

[19] Akbar S.M.F., Al Mahtab M., Aguilar J.C., Yoshida O., Penton E., Guillen G., Hiasa Y. (2021). Sustained antiviral and liver protection by a nasal therapeutic vaccine (NASVAC), containing both HBsAg and HBcAg) in patients with chronic hepatitis B; 2-year follow-up of phase III clinical trial. Pathogens.

[20] Lobaina Y., Palenzuela D., García D., Rodríguez D., Pichardo D., Muzio V., Aguilar J. (2006). Comparative study of the immunogenicity and immunoenhancing effects of two hepatitis B core antigen variants in mice by nasal administration. Vaccine.

[21] Yoshida O., Imai Y., Shiraishi K., Tokumoto Y., Sanada T., Tsukiyama-Kohara K., Miyazaki T., Kamishita T., Aguilar J.C., Guillen G.E. HBsAg Reduction by Nasal Administration of a Therapeutic Vaccine Containing HBsAg and HBcAg (NASVAC) in Patients with Chronic HBV Infection: The Results of 18 Months Follow-Up. Proceedings of the Liver Meeting Digital Experience™.

